# A rare fatal case of adenovirus serotype 4 associated acute disseminated encephalomyelitis in an adult: A case report

**DOI:** 10.1016/j.idcr.2021.e01213

**Published:** 2021-06-30

**Authors:** Zahra Qamar, Catherine M. Tucker, Lawrence C. Kenyon, Tricia L. Royer

**Affiliations:** a Department of Medicine, Division of Infectious Diseases, Thomas Jefferson University Hospital, 1015 Chestnut Street, Suite 1020, Philadelphia, PA, 19107, United States; b Department of Pathology, Anatomy and Cell Biology, Thomas Jefferson University Hospital, 280 Main Building, 132 S. 10th Street, Philadelphia, PA, 19107, United States

**Keywords:** Adenovirus, Acute disseminated encephalomyelitis, ADEM, Adult

## Abstract

Acute disseminated encephalomyelitis (ADEM) is an autoimmune demyelinating disease directed against the myelin sheath of the central nervous system that typically presents 1–4 weeks after an infection or vaccination, most commonly in children. We describe a case of a young female who presented with rapidly progressive mental deterioration and died secondary to ADEM following an adenovirus upper respiratory tract infection.

## Case description

A 27-year-old female was admitted in July 2019 for altered mental status. She was a flight attendant and had returned from Ireland 1 week prior to presentation. Upon return, she noted a sore throat and myalgias for which she took over the counter ibuprofen. The night prior to admission, her partner noticed her moaning and unresponsive and called EMS. Upon admission, she was stuporous. Additional relevant past medical history provided by her family included well-controlled anxiety, not requiring medications. There was no significant surgical or family history and no previous hospitalizations. She never smoked, used alcohol occasionally and did not use recreational drugs. She had moved to Philadelphia from California two months prior. Her travel history was pertinent for multiple work related domestic flights and one international trip to Ireland.

On arrival, she was noted to have a fever of 104 °F, pulse 110 bpm, blood pressure 100/50 mm Hg and oxygen saturation of 99 % on room air. She was well-nourished, without conjunctivitis, pharyngeal erythema, or lymphadenopathy. Skin examination was notable for a faint, erythematous, maculopapular rash on her trunk and neck, sparing her extremities. Neurological examination was non-focal and there was no nuchal rigidity. Complete blood count revealed a white blood cell (WBC) count of 8.7 × 10^9^/l (90 % neutrophils, 3% lymphocytes and 6% monocytes), hemoglobin of 13.0 g/dl and platelet count of 199,000 109/l. Blood chemistry revealed normal electrolytes, hepatic and renal function. A lumbar puncture (LP) showed an opening pressure of 25 cm H_2_O, WBC count 49/μL(neutrophils 12 %, lymphocytes 78 %), RBC count 0, protein 107 mg/dL and glucose 69 mg/dL. A CT scan of the head without contrast revealed no acute abnormality. MRI of the brain without contrast revealed no acute infarction, hemorrhage, mass or abnormal enhancement.

Given the suspicion of bacterial or viral meningitis and/or encephalitis, intravenous vancomycin, ceftriaxone, acyclovir and steroids were empirically started. Cerebrospinal fluid (CSF) gram stain, culture and a CSF PCR Panel (with targets for *H.influenzae, L.monocytogenes, N.meningitidis, S.agalactiae, S.pneumoniae*, Cytomegalovirus, Enterovirus, Herpes simplex virus 1 and 2, Human herpesvirus 6, Human parechovirus, Varicella Zoster virus, *Cryptococcus neoformans/gatti,* and *E.coli K1*) failed to reveal an infectious etiology. Further work up was sent for evaluation of Epstein-Barr virus, West Nile Virus, Tick borne Encephalitis virus, Powassan virus, Eastern Equine Encephalitis virus, *Borrelia burgdorferi,* and *Anaplasma phagocytophilum*, all of which were ultimately negative.

On the second day of hospitalization, the patient's mental status deteriorated rapidly requiring intubation for airway protection. A repeat CT of the head without contrast revealed no acute changes and a repeat LP continued to reveal pleocytosis and elevated protein (WBC 134 cells/μL, protein 84 mg/dL). On hospital day 3, she developed bradycardia, hypertension, and loss of gag and corneal reflexes, indicative of raised intracranial pressure. A CT of the head now revealed generalized cerebral swelling at which time the patient was taken for emergent decompressive hemicraniectomy. A viral respiratory pathogen PCR panel obtained on admission returned positive for adenovirus. Adenovirus PCR was sent from the CSF, blood and bronchoalveolar lavage (BAL). Cidofovir and probenecid were started for suspicion of disseminated adenovirus infection. On hospital day 9, she developed ventilator associated pneumonia due to *Pseudomonas aeruginosa* which was treated with IV cefepime. Her mental status continued to worsen and the family decided to withdraw care on hospital day 10. The adenovirus from the respiratory sample was later identified as Serotype 4. Adenovirus was not detected in the CSF or blood.

A complete autopsy was performed. The gross pathology was remarkable for diffusely congested lungs bilaterally and mild bilateral pleural effusions. Microscopy revealed massive pulmonary edema with severe acute bronchopneumonia. Gross examination of the brain (1280 g) demonstrated cerebral edema with bilateral tonsillar herniation. Multiple microhemorrhages were identified in the bilateral cerebral hemispheres, pons, medulla, and spinal cord. These microhemorrhages were secondary to cerebral edema. In addition, there were multiple dusky areas in the white matter tracts of the cerebral cortex, most prominent on the right, concerning for demyelination.

**Fig. 1:**
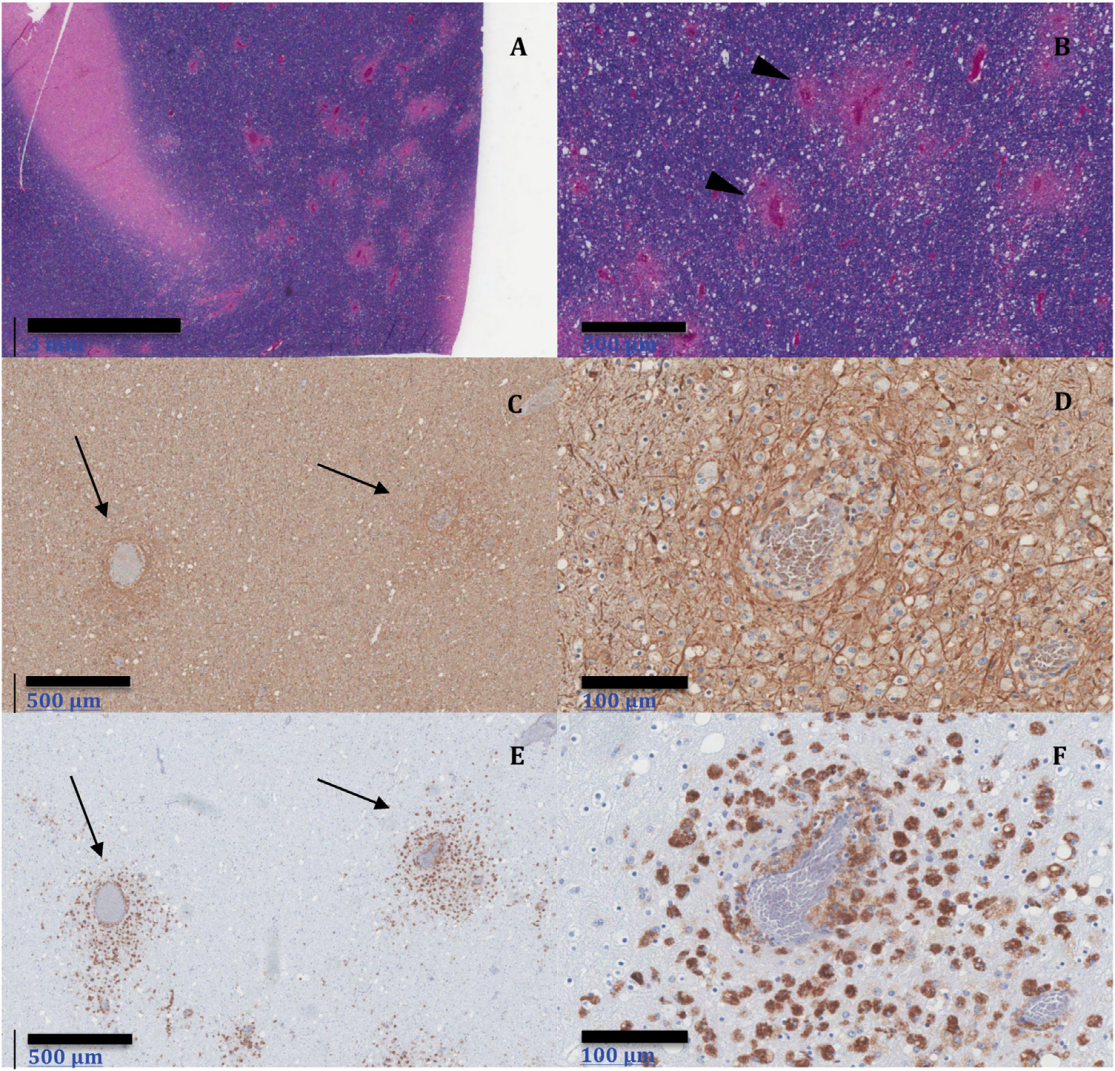
Histologic sections of involved occipital lobe. Luxol Fast Blue/PAS stain, Panel A (20X) and Panel B (40X), showing perivenous demyelination (pink areas around vessels) in areas of macrophage infiltration (arrowheads). Neurofilament protein stain demonstrates axonal preservation (arrows) in these areas, Panel C (40X) and Panel D (100X). Perivenous macrophage infiltration (arrows) is highlighted by CD68 immunohistochemistry, Panel E (40X) and Panel F (100X) (For interpretation of the references to colour in this figure legend, the reader is referred to the web version of this article).

Microscopic examination showed perivascular clusters of macrophages and mild perivascular lymphocytic infiltrates in innumerable discrete foci throughout the cerebral white matter, thalamus, brainstem, and spinal cord. Luxol Fast Blue/Periodic Acid-Schiff (LFB/PAS) stains confirmed the presence of perivascular demyelination (Fig. 1, Panel A and Panel B). Bielschowsky stain and immunohistochemistry for neurofilament protein revealed axonal preservation in areas of demyelination (Fig. 1, Panel C and Panel D). Perivenous macrophage infiltration was confirmed by CD68 and CD163 immunohistochemical stains (Fig. 1, Panel E and Panel F). CD3 and CD8 immunohistochemistry corroborated the presence of perivascular and parenchymal cytotoxic T lymphocytes, which were scant in comparison to the number of macrophages (data not shown). Immunohistochemistry was negative for adenovirus. The neuropathologic evaluation was diagnostic of acute disseminated encephalomyelitis (ADEM). The cause of death was ADEM and severe acute bronchopneumonia.

## Discussion

ADEM, also known as post infectious encephalitis, is an autoimmune demyelinating disease directed against the myelin sheath of central nervous system axons that follows an infection or rarely vaccination. It is most commonly encountered in children and typically develops after a delay of 1–4 weeks [1,2]. Non-specific upper respiratory tract infections have been most frequently associated with the most severe form of the disease, as seen in our patient [3]. Viral infections identified with ADEM include influenza, mumps, HSV encephalitis, enterovirus, Epstein-Barr virus, human herpes virus-6, dengue virus, adenovirus, hepatitis C, and others [[4], [5], [6]]. Bacterial infections including *Chlamydia pneumoniae* and *Mycoplasma pneumoniae*, *Borrelia burgdorferi*, *Rickettsia rickettsii*, among others, have also been implicated [7,8].

While the exact pathogenesis is still unknown, genetic susceptibility may play a role, as demonstrated by an association of ADEM with certain major histocompatibility complex alleles. Some myelin antigens may share structural homology with antigenic determinants present on the pathogen responsible for ADEM. It is postulated that antibodies produced against the infecting pathogen cross-react with myelin antigens, resulting in an immune response against myelin (molecular mimicry). Alternatively, it is hypothesized that CNS infection with neurotropic viruses results in myelin-based antigens entering the systemic circulation through a disrupted blood-brain barrier leading to targeting of these antigens by the immune system. Within days of onset, microglia, lymphocytes, and phagocytes appear, ultimately triggering demyelination in a perivenous pattern [1,8,9]. The most common histopathological finding in ADEM is perivenous infiltration by macrophages. In fact, many reports emphasize the finding of perivenous sleeves of inflammation and demyelination as the pathologic hallmark and predominant lesion of ADEM [9]. Such characteristic features of perivenous demyelination were widespread in the brain and spinal cord of our case. In contrast, multiple sclerosis lesions are typically large confluent areas of demyelination with a paraventricular predilection. As opposed to acute viral encephalitides, attempts to isolate responsible viruses from post-mortem ADEM brains have often failed, implying mechanisms other than direct invasion of the CNS by the infectious agent [3].

Clinical onset is acute and often rapidly progressive. Presenting manifestations are nonspecific, including prodromal symptoms of fever, malaise, headache, nausea or vomiting [3]. Encephalopathy, the main characteristic feature, develops within 7 days of prodromal symptoms. Neurological symptoms may include behavioral changes, confusion, irritability, restlessness and finally obtundation and coma. Focal neurologic deficits may be present, such as visual impairment, hemiparesis, paraparesis, bladder dysfunction and sensory disturbances [8]. Meningismus occurs in 26–31 % of cases [3]. These symptoms are nonspecific, and infectious and autoimmune causes should be excluded prior to making a diagnosis of ADEM. While the majority of cases are monophasic, some patients have recurrent demyelinating episodes which may be confused with multiple sclerosis [8].

Upon diagnostic workup, peripheral blood counts are usually normal, except leukocytosis may be observed. CSF analysis may be normal or exhibit a lymphocytic pleocytosis and elevated protein. This finding, coupled with a preceding infection is suggestive, but not required for diagnosis of ADEM. There are no specific biomarkers or confirmatory tests to establish the diagnosis of ADEM premortem [9]. The best tool for diagnosis is an MRI of the brain which shows multiple new demyelinating lesions. The majority of cases in the literature were associated with abnormal MRI findings, in comparison to our patient, who had a normal brain MRI [3]. This discrepancy is most likely due to imaging at a very early point in the clinical evolution of ADEM in this patient.

Treatment for ADEM is based on case reports and case series as there are no randomized control trials addressing management. High dose glucocorticoids have been the most widely used. Use of intravenous immunoglobulin (IVIG) and plasmapheresis have also been described for steroid refractory patients. Most patients recover completely after steroid therapy. In one case series, the majority recovered or had minor disabilities while 11 % suffered residual neurologic deficits. Recovery is faster and more complete in children as compared to adults [1,3,8].

Our patient was treated with cidofovir and probenecid given the working diagnosis of disseminated adenovirus infection, based on isolation of adenovirus from a respiratory sample. There are no FDA approved antiviral agents for the treatment of adenovirus infections. Cidofovir, along with probenecid to prevent nephrotoxicity, is considered the standard practice for treatment of severe, progressive, or disseminated adenovirus disease in transplant patients and we extrapolated its use in our patient [10].

### Conclusion

In summary, we present a rare case of adenovirus associated ADEM in an adult. ADEM is much more common in children and is not typically fatal in adults [6]. Our 27 year-old patient with no significant medical history presented with rapidly progressive obtundation after a mild upper respiratory adenovirus infection. Classic gross and histologic features of ADEM were present at autopsy. There were no clinical or radiological features of ADEM which could differentiate her presentation from viral encephalitis during life, hence ADEM was not suspected and therapies including high dose steroids, IVIG or plasmapheresis were not utilized. Adenovirus is such a rare cause of ADEM, particularly in adults, that one must maintain a high level of suspicion in order to accurately diagnose and treat this potentially fatal condition.

Authors have no conflicts of interests. All authors listed below have seen and approved the manuscript, and their contributions are listed below. The manuscript has not been previously published and is not being considered for publication elsewhere. Patient consent was not obtained as no patient identifiers were used.
